# Key characteristics of anti-CD20 monoclonal antibodies and clinical implications for multiple sclerosis treatment

**DOI:** 10.1007/s00415-023-12007-3

**Published:** 2023-10-31

**Authors:** Silvia R. Delgado, Simon Faissner, Ralf A. Linker, Kottil Rammohan

**Affiliations:** 1 Department of Neurology, Leonard M. Miller School of Medicine, University of Miami, Miami, FL USA; 2 Department of Neurology, Ruhr-University Bochum, St Josef-Hospital, Bochum, Germany; 3 Department of Neurology, University Hospital Regensburg, Regensburg, Germany

**Keywords:** Multiple sclerosis, Anti-CD20, Ofatumumab, Ocrelizumab, Rituximab, Ublituximab

## Abstract

The recent success of anti-CD20 monoclonal antibody therapies in the treatment of multiple sclerosis (MS) has highlighted the role of B cells in the pathogenesis of MS. In people with MS, the inflammatory characteristics of B-cell activity are elevated, leading to increased pro-inflammatory cytokine release, diminished anti-inflammatory cytokine production and an accumulation of pathogenic B cells in the cerebrospinal fluid. Rituximab, ocrelizumab, ofatumumab, ublituximab and BCD-132 are anti-CD20 therapies that are either undergoing clinical development, or have been approved, for the treatment of MS. Despite CD20 being a common target for these therapies, differences have been reported in their mechanistic, pharmacological and clinical characteristics, which may have substantial clinical implications. This narrative review explores key characteristics of these therapies. By using clinical trial data and real-world evidence, we discuss their mechanisms of action, routes of administration, efficacy (in relation to B-cell kinetics), safety, tolerability and convenience of use. Clinicians, alongside patients and their families, should consider the aspects discussed in this review as part of shared decision-making discussions to improve outcomes and health-related quality of life for people living with MS.

## Introduction

The neurodegenerative and demyelinating characteristics of multiple sclerosis (MS) have historically been viewed as a pathological consequence of aberrant T-cell function [1–4]. However, the more recent success of treatment for MS using anti-CD20 strategies has highlighted the significant role of B cells in the pathogenesis of MS [2–5]. B cells are critical mediators of humoral immunity, and they possess various pro-inflammatory and regulatory characteristics [6, 7]. In MS, the regulatory functions of B cells are altered, and the pro-inflammatory characteristics are heightened [8, 9]. These factors lead to several immunopathogenic mechanisms mediated by B cells, such as:

- The overproduction of pro-inflammatory cytokines, such as interleukin (IL)-6 and tumor necrosis factor alpha [9]
- The reduced production of anti-inflammatory cytokines, such as IL-10 [9]
- The accumulation of pathogenic B cells to the cerebrospinal fluid (CSF) and the subsequent existence of oligoclonal bands within the CSF [10]
- The stimulation of T-cell proliferation by antigen-presenting B cells, and interplay between activated pathogenic and infiltrative B and T cells within germinal centers and the central nervous system (CNS) [1, 11]
- The formation of ectopic lymphoid follicle structures or tertiary lymphoid structures in the meninges [12–14].

The pathological involvement of B cells in MS led to the development and utilization of B cell-depleting therapies that target B cell-specific components: primarily CD20 in MS [15]. CD20 is a transmembrane, non-glycosylated phosphoprotein that is primarily expressed on the cell surface during almost all stages of the B-cell life cycle, except for pro-B cells and terminally differentiated plasmablasts and plasma cells [16]. CD20 is also expressed on a small percentage of circulating T cells [17]. The physiological function and ligand of CD20 are currently unclear; however, it is purported to have roles in the differentiation and growth of B cells, as well as having a role in the modulation of calcium flux [16, 18].

CD20 is not alone as a target for B cell-depleting therapies. Inebilizumab, an anti-CD19 monoclonal antibody (mAb) therapy approved for the treatment of neuromyelitis optica spectrum disorders, is undergoing clinical assessment as a treatment for MS [19, 20]. Unlike CD20, CD19 expression is maintained during the maturation of pro-B cells to plasmablasts, which are believed to play an important part in the CNS inflammation that is characteristic of MS [20–22]. However, there are potential safety concerns associated with the depletion of a broader spectrum of B cells [23].

As such, owing to its comparatively selective presence on the surface of certain populations of B cells, CD20 may present a more attractive target to enable depletion of B cells in B cell-associated cancers and autoimmune diseases, such as MS. For instance, certain anti-CD20 mAbs are currently approved by the US Food and Drug Administration (FDA) and/or European Medicines Agency (EMA) for the treatment of B cell-associated cancers, such as non-Hodgkin lymphoma and chronic lymphocytic leukemia [24–27]. Furthermore, anti-CD20 mAbs are also FDA and/or EMA approved for or undergoing clinical evaluation in several autoimmune diseases, including rheumatoid arthritis [25, 27], systemic lupus erythematosus [28] and polyangiitis [24, 25, 27], as well as various disease courses of MS [29–32].

There are currently five anti-CD20 therapies undergoing clinical development, or that have been approved, for the treatment of MS.

- Rituximab: not currently approved for use in MS but frequently used off-label [33].
- Ocrelizumab (Ocrevus^®^): EMA and FDA approved for a) relapsing forms of MS (RMS) including clinically isolated syndrome (CIS), relapsing-remitting MS (RRMS) and active secondary progressive MS (SPMS) and b) primary progressive MS (PPMS) [29, 31, 34].
- Ofatumumab (Kesimpta^®^): EMA approved for RMS and FDA approved for RMS (CIS, RRMS and active SPMS) [30, 32].
- Ublituximab: EMA approved for RMS and FDA approved for RMS (CIS, RRMS and active SPMS) [35–38].
- BCD-132: undergoing clinical evaluation and not yet approved for use in MS [40].

Despite these MS therapies sharing a common target, differences have been reported in their mechanistic, pharmacological and clinical characteristics, which may have substantial clinical implications and contribute to the respective treatment efficacies of anti-CD20 therapies [41]. There are several measures of MS treatment efficacy, such as annualized relapse rates, disability progression and magnetic resonance imaging measures of disease progression, which have been discussed in prior reviews of anti-CD20 therapies for MS [42–44]. Clinicians should take these implications into consideration during treatment decision-making processes to improve outcomes and health-related quality of life for people living with MS.

This review will compare and contrast the characteristics of the five anti-CD20 mAbs in MS and discuss the clinical implications of these differences, with a particular focus on their mechanisms of action, routes of administration, efficacy (in relation to B-cell kinetics), safety, tolerability and convenience of use.

## Evidence identification

Literature for this review was identified through a provisional scoping search, followed by targeted literature searches through PubMed, the Embase database via the Ovid platform and ClinicalTrials.gov. Sources included in this review comprised original peer-reviewed research articles, review articles, expert opinion articles, conference presentations and posters, and publicly available clinical trial data. This review adhered to the Scale for the Assessment of Narrative Review best practice guideline for the development of narrative reviews [45]. Literature searches focused on mechanism of action, route of administration, efficacy, safety, tolerability and convenience of use of the five anti-CD20 mAbs identified during the provisional scoping search. Search terms included “multiple sclerosis,” “anti-CD20,” “ofatumumab,” “ocrelizumab,” “rituximab,” “ublituximab” and “BCD-132.” The titles and/or abstracts of search results were screened by a single reviewer for relevance to this review in October 2022.

## Mechanism of action and route of administration

### Molecular structure and mechanism of action

Anti-CD20 mAbs are classified into two distinct types—type I or II—depending on whether the CD20-bound antibody induces reorganization of CD20 molecules into cell-surface lipid rafts [46–48].

- Type I: reorganization of CD20 molecules into lipid rafts and activation of the complement system pathway, resulting in high complement-dependent cytotoxicity (CDC).
- Type II: no reorganization of CD20 molecules into lipid rafts and poor induction of CDC. However, type II antibodies can potently induce direct, non-apoptotic cell death by binding to CD20.

The disparate characteristics of type I and II anti-CD20 mAbs do not translate into any differences in their ability to induce antibody-dependent cellular cytotoxicity (ADCC) or antibody-dependent cell-mediated phagocytosis (ADCP). These immune responses are mediated by interactions between the antibody’s Fc region and FcγRIIIa molecules on the surface of immune effector cells [46, 48].

All of the antibodies either approved for or undergoing clinical evaluation for MS in this review are type I antibodies [36, 48]. Despite not having undergone extensive clinical evaluation in MS, preclinical data suggest that type II antibodies, such as tositumomab and obinutuzumab, may have future clinical utility for people with MS owing to their ability to deplete B cells in blood and lymphoid tissue more efficiently than type I antibodies [49–51]. However, these preclinical results are not disease specific and have yet to be translated into clinical evaluations in people with MS. Therefore, this review will focus on type I anti-CD20 mAbs in the treatment of MS.

### CD20 and anti-drug antibodies

Rituximab and ublituximab are chimeric immunoglobulin (Ig)G_1_ antibodies and are therefore more likely to induce anti-drug antibodies (ADAs) than fully human or humanized antibodies (Table 1) [36, 52]. This appears to be the case with rituximab, with 7.0% (20/286) and 24.1% (14/58) of patients testing positive for anti-chimeric antibodies in the phase II/III OLYMPUS trial in PPMS and phase II HERMES trial in RRMS, respectively [52, 53]. Relatively high levels of anti-chimeric antibodies have also been reported in a phase I rituximab trial in RRMS by Bar-Or et al*.* (28.6% [6/21]), as well as in trials of rituximab in other autoimmune indications, such as rheumatoid arthritis and polyangiitis [25, 54]. For ublituximab, results from the phase III ULTIMATE trials in RMS reported 81.3% (434/534) and 5.8% (31/534) of patients developing ADAs and neutralizing antibodies (NAbs), respectively.

**Table 1:**
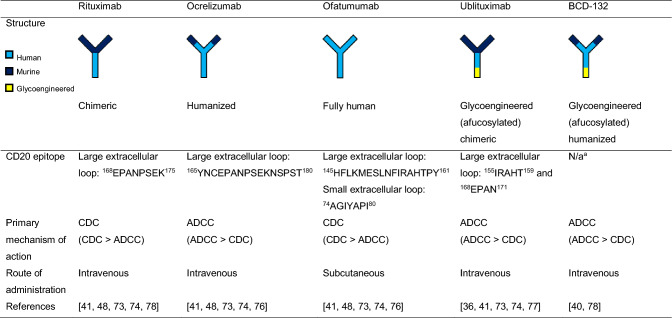
Structures, epitopes, mechanisms of action and routes of administration of anti-CD20 monoclonal antibodies approved for or under clinical investigation for use in MS

^a^Information is not currently publicly available

*ADCC* antibody-dependent cellular cytotoxicity, *CD* cluster of differentiation, *CDC* complement-dependent cytotoxicity, *MS* multiple sclerosis, *N/a* not available

Ocrelizumab, as a humanized IgG_1_, is expected to have greater immunogenicity than ofatumumab but lower immunogenicity than the chimeric antibodies rituximab and ublituximab (Table 1) [55]. Indeed, the ORATORIO phase III trial in PPMS reported that 1.9% (9/486) of patients had ADAs and 0.2% (1/486) had NAbs [55]. Similarly, the phase III OPERA I and II trials in RMS reported 0.4% (3/825) and 0.1% (1/825) of patients developing ADAs and NAbs, respectively [56].

Ofatumumab is currently the only fully human IgG_1_ that is approved for use in all RMS disease courses as a low-dose subcutaneous injection [30, 32] (Table 1). Likely as a result, ofatumumab has lower immunogenicity with a lower potential to induce ADAs than the other mAbs. In the phase III ASCLEPIOS I and II studies of ofatumumab in RMS, ADAs developed in 0.2% (2/946) of patients, with no patients developing NAbs [57]. Furthermore, trials of ofatumumab in differing indications, such as chronic lymphocytic leukemia, with increased dosages (up to 1000 mg) and intravenous administration have also shown very low incidences of ADAs and NAbs [26, 58].

The biological and clinical implications of ADAs on efficacy and safety are uncertain, with no conclusive evidence that development of such antibodies has a detrimental effect on either efficacy or safety [52, 53, 59]. However, previous case study reports have suggested a potential role in the development of serum sickness following treatment with rituximab or ocrelizumab [60, 61]. It should also be noted that reporting of ADA positivity across trials may be affected by several factors, such as sample preparation, assay characteristics, drug interference, concomitant medications and MS disease course; therefore, cross-trial comparisons may be misleading [31, 41, 62]. The ADA data for humanized BCD-132 are currently unavailable. Given that ADAs can be non-neutralizing [63], the development of NAbs may be of greater clinical significance owing to impaired pharmacological function of the drug. It may therefore be beneficial to monitor NAb level to avoid anti-CD20 treatment failure.

### Comparison of CDC versus ADCC properties

The differing molecular structures and binding epitopes of the anti-CD20 mAbs also substantially contribute to differences in their primary mechanisms of action (Table 1). It is widely agreed that the major mechanisms of B-cell depletion by type I antibodies occur through CDC and ADCC, with relatively minor contributions from ADCP and direct cell death [41].

**Fig. 1:**
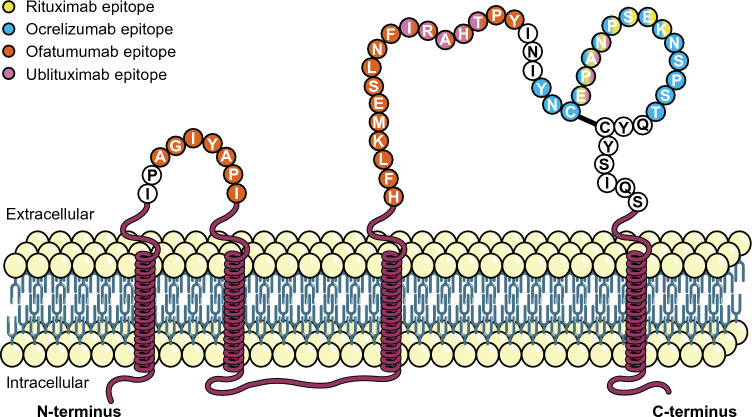
Binding epitopes of anti-CD20 monoclonal antibodies approved for or under clinical investigation for use in MS [36, 41, 48]. The binding epitope of BCD-132 is not currently publicly available. Adapted from Fox et al. and Bar-Or et al. [36, 41]. *CD* cluster of differentiation, *MS* multiple sclerosis

Ocrelizumab and rituximab both target highly similar and overlapping epitopes of CD20 around amino acid residues 165–180 on the large extracellular loop (Table 1; Fig. 1) [41, 48]. Despite this, ocrelizumab’s primary mechanism of B-cell depletion is through ADCC with minor CDC contribution, while rituximab primarily induces greater levels of CDC than ADCC [64]. These differences in primary mechanism of action are thought to stem from differences in the Fc regions of these two antibodies, with ocrelizumab displaying greater binding to low-affinity variants of FcγRIIIa [64, 65].

Ofatumumab targets a distinct epitope from rituximab and ocrelizumab, overlapping with part of the ublituximab epitope. In addition to targeting a region of the large extracellular loop located N-terminally to the rituximab/ocrelizumab epitope, it also targets a 7-residue region in the small extracellular loop (Table 1; Fig. 1) [41, 48]. This epitope is thought to account for ofatumumab’s closer and tighter binding to CD20, as well as a slower off-rate, than rituximab and ocrelizumab [66–69]. These factors have been hypothesized to result in the observed enhanced CDC compared with the other antibodies because of more efficient binding and deposition of complement system components at the target cell surface [48, 66, 70–72]. This is in addition to ofatumumab’s mediation of CDC being less dependent on cell-surface density, which is usually a critical factor for inducing effective CDC, than rituximab's [70].

Ofatumumab’s greater CDC than rituximab, ocrelizumab and ublituximab [66, 67, 71, 73, 74] is thought to mediate its greater efficacy at lower doses and suitability for subcutaneous administration compared with rituximab, ocrelizumab and ublituximab [67, 68]. Furthermore, although CDC is ofatumumab’s primary mechanism of action, ofatumumab has also been shown to induce twofold higher ADCC than rituximab, with levels similar to ocrelizumab and reduced compared with ublituximab (Table 1) [41, 75, 76].

Ublituximab’s epitope spans two distinct regions of the large extracellular loop, which overlap with the disparate ofatumumab and ocrelizumab/rituximab large extracellular loop epitopes (Table 1; Fig. 1), and exhibits far greater ADCC than CDC [36, 41, 48]. Ublituximab has been shown to induce greater levels of ADCC than rituximab, ocrelizumab and ofatumumab [41]. This is primarily due to ublituximab’s glycoengineered Fc region, which has been afucosylated to enhance FcγRIIIa binding and recruitment of immune effector cells [77]. The epitope for BCD-132 has not yet been publicly reported. However, it has been shown to induce greater levels of ADCC than rituximab (probably owing to Fc region afucosylation) and comparable levels of CDC (Table 1) [40, 78]. It must be noted that those mAbs that primarily function via ADCC (ublituximab, ocrelizumab and BCD-132) are particularly prone to Fcγ receptor polymorphisms. Such polymorphisms should be clinically considered, as their presence may have profound effects upon treatment efficacy [79–82].

### Effect of B-cell depletion on T-cell function

Recent studies have also highlighted important treatment-induced effects on T-cell populations. While CD20 is predominantly expressed on B cells, a small proportion of T cells also express CD20. The function of these CD3+ CD20+ T cells remain unknown. Direct targeting and depletion of CD20+ T cells and indirect effects upon T-cell populations stemming from abrogated B:T-cell interactions as a result of treatment-induced B-cell depletion have been observed [83–87]. Hematological analysis found that CD3+ CD20+ T cells formed 2.4 ± 0.36% (mean ± standard error of the mean [SEM]) of all CD45+ lymphocytes in patients with MS before ocrelizumab treatment. Two weeks post-treatment, CD3+ CD20+ T cells formed 0.04 ± 0.01% (mean ± SEM) of all CD45+ lymphocytes, indicating that ocrelizumab can rapidly diminish CD20+ T-cell numbers [86]. Similarly, patients treated with rituximab had almost complete loss of CD3+ CD20+ T cells over 12 weeks [85]. Likewise, blood samples taken from patients with MS before and after ublituximab treatment showed that the percentage of total T cells declined from 45–29% 24 h after treatment [87]. In patients with RRMS, ofatumumab was found to increase control of effector T cells, decrease T-cell autoreactivity, reduce peripheral CD20+ T cells and decrease the migratory capacity of T cells, when compared with treatment-naive patients [83]. Further work by Faissner et al. reported ofatumumab-induced modulation of regulatory T cells with reduced non-suppressive regulatory T cells, increased naive regulatory T cells and a reduced ratio of circulating T follicular helper cells to circulating T follicular regulatory cells in patients with MS compared with treatment-naive healthy controls [84]. Similar effects upon T-cell populations have also been reported for ocrelizumab [86]. The clinical significance of depleting CD20+ T cells is not yet fully understood; however, it has been proposed as a mechanism that may contribute to the efficacy of anti-CD20 mAb therapies [85–87].

### Route of administration

The majority of anti-CD20 mAbs for MS are administered via intravenous infusion, with the subcutaneous injection of ofatumumab being the exception. Studies have shown that these different routes of administration have biological and clinical consequences for the treatment of B cell-associated disease, including MS. For instance, subcutaneous administration of anti-CD20 therapies in mice has been shown to result in similar levels of depletion of circulating and lymph-node-localized CD20+ B cells when administered at a low dose, compared with the high-dose intravenous administrations [76]. Furthermore, low-dose subcutaneous administration is clinically associated with reduced administration-related reactions [41].

Subcutaneously administered anti-CD20 mAbs have a different antibody distribution and localization when compared with intravenous administration. For example, subcutaneous administration resulted in direct and increased penetration of anti-CD20 mAb into the axillary, subiliac, sciatic and inguinal lymph nodes, as well as CNS components, in murine models of experimental autoimmune encephalomyelitis, compared with intravenous administration [76, 88]. Subcutaneous administration also limits transportation of the antibody into the capillaries, and they instead enter systemic circulation via an indirect route through the lymphatic system [69]. Potentially as a result of more direct lymph-node targeting, subcutaneous administration has also been shown to significantly decrease pathological T-cell infiltration in the brains of murine models of MS, when compared with intravenous administration [69, 76]. The mechanistic implications of direct lymph-node targeting, where pathogenic B and T cells interact before migrating to the brain, through subcutaneous injection may contribute to lower required dosing for ofatumumab and a faster B-cell depletion effect, when compared with intravenous treatments [69, 76, 89].

## Mediators of anti-CD20 mAb efficacy

Clinical trials have revealed that anti-CD20 mAbs significantly improve disease-relevant clinical and imaging outcomes versus placebo or active comparators [35, 36, 52, 53, 55–57, 90–93]. However, differences in B-cell depletion and repletion kinetics exist between the different therapies, and these result in important clinical differences that should be considered by neurologists.

### B-cell depletion

Clinical trials of rituximab in MS utilizing two 1000 mg administrations separated by 2 weeks have shown rapid and efficient B-cell depletion characteristics (Table 2). The phase II/III OLYMPUS trial reported rapid and > 95% depletion of CD19+ B cells 2 weeks after the first 1000 mg infusion, and this low level persisted through to the end of the study (week 96) [53]. This mirrored earlier observations (Table 2) [52, 54]. In the phase III ORATORIO trial of intravenous ocrelizumab 600 mg administered every 24 weeks (Table 2), CD19+ B-cell populations were almost completely depleted 2 weeks after the first 600 mg administration and remained at this level for the remainder of the 216-week trial [55]. Consistent B-cell depletion kinetics after 2 weeks on study were reported in the phase III OPERA trials, which utilized a similar dosing regimen (Table 2) [56].

**Table 2:** Key clinical trials of anti-CD20 therapies showing dose, route of administration, reported B-cell depletion and administration-related reaction data for each different monoclonal antibody

Study	Dose(s) and RoA	B-cell depletion kinetics data	IRR data
Rituximab^a^			
HERMES [52] (NCT00097188)	2 doses of RTX 1000 mg i.v. 2 weeks apart, or placebo	RTX B-cell depletion 2 weeks after first 1000 mg infusion: > 95% depletion of CD19+ B cells RTX maintained low B-cell levels through to week 24 (EoS)	RTX IRRs: 78.3% (54/69) Placebo IRRs: 40.0% (14/35)
OLYMPUS [53] (NCT00087529)	2 doses of RTX 1000 mg i.v. 2 weeks apart repeated q24w,^b^ or placebo	RTX B-cell depletion 2 weeks after first 1000 mg infusion: > 95% depletion of CD19+ B cells RTX maintained low B-cell levels through to week 96 (EoS)	RTX IRRs (after first infusion): 67.1% (196/292) Placebo IRRs (after first infusion): 23.1% (34/147)
Bar-Or et al. [54]	2 doses of RTX 1000 mg i.v. 2 weeks apart, repeated at week 24	RTX B-cell depletion 2 weeks after first 1000 mg infusion: 99.8% depletion of CD19+ B cells RTX maintained low B-cell levels through to week 48 (EoS)	RTX IRRs: 65.4% (17/26)
Zecca et al. [107]	Various—see footnote	Data not available	RTX IRRs (mild or moderate): 46.1% (146/317) RTX IRRs (serious): 4.4% (14/317)
Ocrelizumab^c^			
ORATORIO [55] (NCT01194570)	OCR 600 mg q24w i.v.,^d^ or placebo	OCR B-cell depletion 2 weeks after first 600 mg infusion: almost complete depletion of CD19+ B cells OCR maintained low B-cell levels through to week 216 (EoS) Placebo had negligible effect on CD19+ B-cell levels	OCR IRRs: 39.9% (194/486) Placebo IRRs: 25.5% (61/239)
OPERA I and II [56] (NCT01247324/NCT01412333)	OCR 600 mg q24w i.v.,^e^ or IFN 44 μg tiw s.c	OCR B-cell depletion 2 weeks after first 600 mg infusion: almost complete depletion of CD19+ B cells OCR maintained low B-cell levels through to week 96 (EoS) IFN had substantially decreased and temporal effect on CD19+ B-cell levels	OCR IRRs: 34.3% (283/825) IFN IRRs: 9.7% (80/826)
Ofatumumab^f^			
ASCLEPIOS I and II [57] (NCT02792218/NCT02792231)	OMB 20 mg q4w s.c.,^g^ or TFL 14 mg p.o. qd	OMB B-cell depletion 2 weeks into treatment: > 95% of patients below LLN (40 cells/μL) and 82% ≤ 10 cells/μL OMB maintained B-cell levels below LLN at all visits from week 2 to EoS at week 120 TFL B-cell depletion throughout study: > 5% of patients below LLN	OMB injection-related systemic reactions^h^: 20.2% (191/946) OMB injection-site reactions: 10.9% (103/946) TFL injection-related systemic reactions^h^: 15.0% (140/936) TFL injection-site reactions: 5.6% (52/936)
APLIOS [94] (NCT03560739)	OMB 20 mg q4w s.c.^g^	OMB B-cell depletion 2 weeks into treatment: 100% of patients below LLN (80 cells/μL) and 84.6% ≤ 10 cells/μL OMB maintained B-cell levels at 1.0 cells/μL at all visits from week 4 to EoS at week 12	OMB injection-systemic reactions: 28.5% (81/284) OMB injection-site reactions: 10.2% (29/284)
APOLITOS [91] (NCT03249714)	OMB 20 mg q4w s.c.,^g^ or placebo	OMB B-cell depletion 1 week into treatment: all patients below LLN (80 cells/μL) B-cell levels maintained in all patients through to week 24	OMB injection-related reactions: 25.0% (10/40) (week 48 analysis) Placebo injection-related reactions: 21.0% (4/19) (week 48 analysis)
OMS115102 [93] (NCT00640328)	2 doses of OMB 100, 300 or 700 mg i.v. 2 weeks apart, or placebo	OMB median B-cell level 1 week into treatment: 0 cells/μL	i.v. OMB IRRs: 78.9% (30/38)
MIRROR [92] (NCT01457924)	OMB 3, 30 or 60 mg q12w s.c., or 60 mg q4w s.c., or placebo	OMB 60 mg q4w B-cell depletion: < 2% of baseline at max. depletion (30/60 mg q12w ~ 5% of baseline; 3 mg q12w ~ 25% of baseline)	Higher-dose OMB IRRs: 52.4% (86/164) Placebo IRRs: 14.9% (10/67)
Ublituximab^i^			
ULTIMATE I and II [35] (NCT03277261/NCT3277248)	UTX 450 mg q24w i.v.,^j^ or TFL 14 mg p.o. qd	UTX B-cell depletion 24 h after first infusion: 96% decrease UTX B-cell depletion through DB period: 97% decrease TFL B-cell depletion 24 h after first infusion: 53% increase TFL B-cell depletion through DB period: 18% increase	UTX IRRs: 47.7% (260/545) TFL IRRs: 12.2% (67/548)
Fox et al. [36] (NCT02738775)	UTX 150 mg on day 1, 450 or 600 mg on day 15 and 450 or 600 mg on week 24 i.v., or placebo (crossover to UTX at week 4)	UTX B-cell depletion 24 h after first infusion: decrease from baseline mean level of 7.3% to 0.2% UTX B-cell depletion 4 weeks into treatment: 100% of patients with ≥ 95% B-cell depletion Maintenance of B-cell reduction through 48-week study, with no significant B-cell repletion	UTX IRRs: 50.0% (24/48) Placebo IRRs: 0% (0/12)
BCD-132			
Boyko et al. [40] (NCT03551275)	BCD 100, 250, 500 or 1000 mg i.v. either at a single visit or over two visits separated by 14 days	BCD-132 B-cell depletion 48 h after infusion of all doses: median CD19+ B-cell level of 0.0% (baseline level of 8.4%)	BCD IRRs: 25.0% (6/24)

Zecca et al. RTX administration: initial dosing either two 375 mg/m^2^ infusions 15 days apart, two 1000 mg infusions 15 days apart, or four 375 mg/m^2^ infusions every week for 4 weeks. Maintenance dosing either fixed time point (6 months) reinfusions of 100 mg 15 days apart or cytofluorimetric-based reinfusion regimens based on CD19+ or CD27+ cells reappearance

^a^Administered as an infusion over the course of 4–6 h for the initial infusion, potentially dropping to 3–4 h with subsequent infusions and must be preceded by premedication 30 min before each infusion

^b^Initial dose given on days 1 and 15, with subsequent q24w injections starting at week 24

^c^Administration of ocrelizumab must be preceded by premedication use 30–60 min before each i.v. infusion. Each of the initial ocrelizumab infusions lasts ≥ 2.5 h, with subsequent infusions lasting either ≥ 2 or 3.5 h. This difference in subsequent infusion duration is based on whether the patient presented with any previous serious IRRs following ocrelizumab administration

^d^Given as two 300 mg infusions separated by 2 weeks

^e^Initial doses of 300 mg i.v. on days 1 and 15, with subsequent 600 mg q24w infusions starting at week 24

^f^Administered as a self-administered s.c. injection via either pre-filled syringe or Sensoready^®^ autoinjector pen

^g^Following an initial loading dose regimen of 20 mg s.c. injections on days 1, 7 and 14, with subsequent q4w injections starting at week 4

^h^Defined as systemic reactions happening ≥ 24 h after any injection

^i^Administered as an initial 4-h infusion on day 1, followed by a 1-h infusion 2 weeks later and subsequent 1-h infusions every 24 weeks thereafter (starting 24 weeks after the initial infusion). Patients must take premedication approximately 30–60 min before each ublituximab infusion and must be monitored for 1 h after the first two infusions by healthcare professionals

^j^Following an initial loading dose regimen of 150 mg i.v. infusion on day 1 and 450 mg i.v. infusion on day 15, with subsequent q4w injections starting at week 24

*BCD* BCD-132, *CD* cluster of differentiation, *DB* double-blind, *EoS* end of study, *IFN* interferon beta-1a, *IRR* infusion-related reaction, *i.v.* intravenously, *LLN* lower limit of normal, *OCR* ocrelizumab, *OMB* ofatumumab, *p.o.* by mouth, *q4w* every 4 weeks, *q12w* every 12 weeks, *q24w* every 24 weeks, *RoA* route of administration, *RTX* rituximab, *s.c.* subcutaneously, *TFL* teriflunomide, *tiw* three times a week, *UTX* ublituximab

The ASCLEPIOS, APLIOS and APOLITOS trials of subcutaneous ofatumumab 20 mg, which all used the same treatment regimen and loading doses (Table 2), reported rapid, efficient and sustained B-cell depletion after 1–2 weeks [57, 90, 91, 94]. In the phase III ASCLEPIOS trials, > 95% of assessed patients had B-cell levels below the lower limit of normal (LLN) at all visits from week 2 until the end of the study (week 120). Similarly, after 2 weeks of ofatumumab treatment, all patients with available data in the phase II APLIOS trial reported B-cell levels below the LLN (80 cells/μL) (Table 2). Finally, during the phase II APOLITOS study, B-cell depletion to below the LLN (80 cells/μL) was observed for all patients on day 7 and was maintained through to week 24 [90].

In a phase II trial of intravenous ublituximab by Fox et al*.*, CD19+ B cells were depleted in most patients within 24 h of the first ublituximab infusion (150 mg), with mean B-cell levels significantly reduced from a baseline level of 7.3% to 0.2% at the 24-h post-initial-infusion time point (Table 2). No significant B-cell repletion was observed, and B-cell reduction levels were sustained for the remainder of the 48-week study [36]. In the larger phase III ULTIMATE trials, patients treated with ublituximab displayed a 96% reduction in the median number of CD19+ B cells after 24 h following the first dose (150 mg) [35]. BCD-132 B-cell depletion data are limited; however, Boyko et al*.* reported a median CD19+ B-cell level of 0.0% (baseline CD19+ levels: 8.4%) 48 h after administration of BCD-132 at a range of doses (100–1000 mg).

B-cell depletion is consistently potent among the trialed and approved dosing regimens for anti-CD20 mAbs, which result in rapid, almost complete abrogation of B cells. It is currently unclear whether such complete depletion is necessary for efficacious therapy for patients with MS, or whether similar levels of efficacy could be achieved with reduced B-cell depletion and therefore reduced dosing [41].

### B-cell repletion

**Fig. 2:**
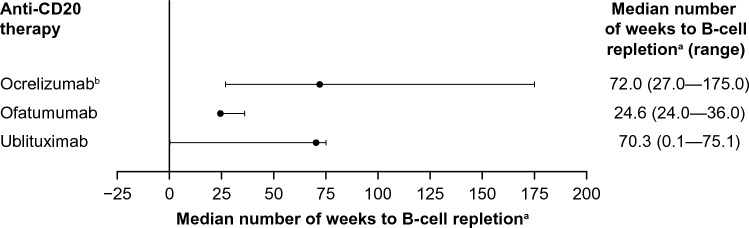
Median number of weeks for B-cell repletion for ocrelizumab, ofatumumab and ublituximab. ^a^Repletion defined as recovery of CD19+ B cells to above the LLN or returned to baseline levels [30, 31, 37, 96]. ^b^Data reflect overall B-cell repletion. *LLN* lower limit of normal

While a consistent and full B-cell depletion is maintained during treatment, faster B-cell repletion has been reported to occur when treatment is discontinued with subcutaneous ofatumumab versus other anti-CD20 therapies, as well as when compared with intravenous ofatumumab [41, 93, 95, 96]. B cells have been shown to recover over the LLN (40 cells/μL), or baseline, in ≥ 50% of patients in 24–36 weeks (median 24.6 weeks) following discontinuation of subcutaneous ofatumumab (20 mg) [95, 97] (Fig. 2). Pharmacokinetic B-cell modeling and simulation for B-cell repletion corroborate this, predicting a median time of 23 weeks [89]. A direct comparison by Savelieva et al*.* reported median repletion times to the LLN, or baseline, for other anti-CD20 mAbs, such as ocrelizumab (72 weeks [range 27–175 weeks]) and rituximab (12–16 months in patients with rheumatoid arthritis) [31, 96, 98] (Fig. 2). A similar median time for CD19+ B-cell counts to return to baseline or the LLN was reported for ublituximab (70.3 weeks [range 0.1–75.1 weeks]) [37] (Fig. 2). Repletion rates for subcutaneous ofatumumab 20 mg were also faster than those observed with intravenous infusion of high-dose (100, 300 or 700 mg) ofatumumab, with only a few patients reporting recovery to above the LLN (100 cells/mm^3^) after 48 weeks [93]. All patients in the intravenous ofatumumab study reported B-cell repopulation within 104 weeks after treatment cessation [93]. The OLYMPUS trial showed that 26 weeks after cessation of rituximab therapy, which is approximately the median time for 50% of patients treated with ofatumumab to reach the LLN (or baseline), a lower proportion of 35% of patients treated with rituximab reached this threshold [53]. Full repletion kinetics data are not currently publicly available for BCD-132.

The characteristic of faster B-cell repletion observed with subcutaneous ofatumumab could be advantageous in some clinical circumstances allowing for flexibility, such as when patients experience infections, require vaccinations or need to restart therapy as quickly as possible following temporary discontinuation [95]. Importantly, B-cell repletion with ofatumumab has not been observed between doses owing to the more regular but low-dose administration regimen compared with other anti-CD20 therapies [89]. However, partial B-cell repletion between half-yearly infusions of ocrelizumab has been reported, but the clinical implications of this are currently uncertain. A study conducted by Toorop et al. was unable to find an association between elevated B-cell levels and the so-called ocrelizumab “wearing-off” phenomenon [99].

### Impact of body weight on B-cell dynamics

In other non-MS indications, anti-CD20 mAb dosages are often given in proportion to a patient’s body weight. Body weight variability with a fixed dose administration can lead to variability in pharmacokinetics and pharmacodynamics in a population, with subsequent differences in therapeutic exposure and resultant efficacy [100]. It is therefore important to determine whether B-cell depletion and repletion kinetics are significantly affected by patient body weight.

In a study of rituximab in rheumatoid arthritis, a relationship between therapy exposure and weight-adjusted administration was observed; however, the association was weak and did not justify adjustments of dose based on patient weight [101]. In MS, a population pharmacokinetic study of ocrelizumab found lower exposure to therapy was predicted with a body-weight-adjusted dose compared with a fixed dose [102]. A study by Turner et al. analyzing subgroup efficacy data from the OPERA I and II trials found that increased ocrelizumab exposure may reduce MS disability progression [103]. When looking at confirmed disability progression at 12 and 24 weeks, the magnitude of ocrelizumab treatment benefit was greater in patients with a lower body mass index (BMI) of < 25 kg/m^2^, who had higher exposure to ocrelizumab, compared with patients with a higher BMI (BMI > 25 kg/m^2^). However, when looking at new or enlarged T2 lesions as an alternative endpoint, the magnitude of ocrelizumab treatment benefit was greater in patients with a higher BMI compared with those with a lower BMI [103].

Pharmacokinetic B-cell modeling of ofatumumab B-cell depletion kinetics with 20 mg subcutaneous administration found that B-cell depletion was relatively independent of the effect of body weight on pharmacokinetics, and B-cell depletion was maintained in patients irrespective of weight [89]. This study did, however, report an association between a longer B-cell repletion time to the LLN (40 cells/μL) and decreasing weight (110 kg, 128 days; 70 kg, 164 days; 50 kg, 204 days) [89]. Finally, analysis of patient data gathered during the ASCLEPIOS I and II trials of ofatumumab did not find a discernible association between variability in body weight and differences in treatment efficacy, based on progression apparently independent of relapse activity [104, 105]. Taken together, the available data suggest that an analysis of multiple efficacy endpoints may be warranted before drawing conclusions on the relationship between body weight and treatment efficacy. A more rigorous analysis could have dosing implications for certain anti-CD20 mAb therapies.

## Safety considerations

The use of anti-CD20 mAb therapies in MS has generally been associated with relatively benign safety profiles [42]. However, there are safety considerations concerning the administration of these therapies, particularly pertaining to administration-related reactions and other immune responses to therapy, with some notable variability between the different antibodies.

### Administration-related reactions

Infusion- and injection-related reactions (IRRs) are the most common adverse events (AEs) observed with administration of anti-CD20 mAbs in people with MS. Signs and symptoms associated with IRRs generally occur within the first 24 h following administration and predominantly after the first administration [41].

Immediate IRRs are common for rituximab and tend to reduce in frequency with subsequent infusions [106]. Data from phase I and II trials of rituximab in MS, as well as real-world evidence data for off-label rituximab use by Zecca et al., reported an incidence of IRRs ranging from 50.5% (160/317) to 78.3% (54/69), with the majority of these AEs being mild to moderate in severity (Table 2) [52–54, 107]. Other reports suggest that severe IRRs develop in approximately 10% of patients treated with rituximab [106]. Severe IRRs typically occur 30–120 min after rituximab infusion and can be fatal [25]. During the Hermes trial, 92.6% (50/54) of participants experienced mild or moderate (grade 1 or 2) IRRs, with 7.6% (4/54) of participants experiencing severe IRRs (grade 3) [52]. Likewise, grade 3 IRRs were reported in 5.8% (17/292) of patients treated with rituximab during the OLYMPUS trial [53].

During the phase III ORATORIO and phase III OPERA trials, IRRs were reported by 39.9% (194/486) and 34.3% (283/825) of patients treated with ocrelizumab, respectively (Table 2) [55, 56]. Within these trials, incidences were compared with the IRR incidences in placebo only (25.5% [61/239]) or placebo-infused interferon beta-1a-treated (9.7% [80/826]) cohorts, respectively (Table 2) [55, 56]. Two patients treated with ocrelizumab in the ORATORIO trial withdrew from the study owing to serious IRRs, while one patient treated with ocrelizumab withdrew from the OPERA trial following a serious IRR of bronchospasm [55, 56]. Subsequent trials have not revealed substantially differing IRR safety profiles, including those evaluating faster ocrelizumab administration compared with the conventional infusion duration (3.5 h), and real-world evidence data have further corroborated the IRR incidence data from the pivotal ORATORIO and OPERA trials [108–114].

In the ASCLEPIOS phase III trials, injection-related systemic reactions (IRSRs) (defined as systemic reactions happening ≥ 24 h after any injection) were observed in 20.2% (191/946) of patients who received subcutaneous ofatumumab, versus 15.0% (140/936) of those who received placebo injections as part of their oral teriflunomide regimen (Table 2) [57]. Of the 191 IRSRs in patients treated with ofatumumab, two were considered serious, with one patient leaving the study after the first injection as a result. Injection-site reactions (ISRs) were reported in 10.9% (103/946) of patients treated with ofatumumab—one of these AEs was serious—versus 5.6% (52/936) of patients treated with teriflunomide (Table 2) [57]. Similarly, the phase II MIRROR study of subcutaneous ofatumumab reported IRRs as the most common AEs in patients treated with ofatumumab (52.4% [86/164] vs. 14.9% [10/67] with placebo injections; Table 2); however, patients were treated with higher doses (30 and 60 mg) of ofatumumab in this trial. Three of these IRRs in patients treated with ofatumumab were considered serious, including one report of cytokine-release syndrome, with all three patients remaining in the trial [92]. Although still being the most commonly reported AE (IRSR, 28.5% [81/284]; ISR, 10.2% [29/284]), there were no serious IRRs in the phase II APLIOS bioequivalence study of subcutaneous ofatumumab 20 mg (Table 2). One patient was reported to have a grade 3 severe reaction after their first pre-filled syringe injection into the abdomen; however, this patient completed the study [94]. The incidence of severe IRRs in < 1.0% of participants during the APLIOS (1/292) and ASCLEPIOS (2/946) studies is lower than the incidence reported in clinical trials of rituximab [52, 53] and ublituximab [74]. The incidence of IRRs in the phase II APOLITOS study was also consistent with the safety profile observed in the other completed studies (week 48 IRR analysis, 25.0% [10/40]) (Table 2) [90, 91]. Interestingly, subcutaneous administration of low-dose ofatumumab resulted in a reduced incidence of IRRs (23.2% [435/1873]) when compared with high-dose ofatumumab administered intravenously (78.9% [30/38]) [93, 115]. Furthermore, interim data from ongoing studies of subcutaneous ofatumumab 20 mg, including the long-term phase IIIb ALITHIOS study [116, 117], have shown consistent IRR profiles, in line with the pivotal studies outlined above.

In the phase III ULTIMATE trials of ublituximab, 47.7% (260/545) of patients reported IRRs with ublituximab, compared with 12.2% (67/548) of teriflunomide-stratified patients who received placebo infusions (Table 2). Grade 3 or higher IRRs were reported in 2.8% of participants. Two patients in this trial experienced grade 4 IRRs, and six patients withdrew from the study owing to serious IRRs [35]. Similar incidences were observed in the phase II study by Fox et al*.*, with 50% (24/48) of patients treated with ublituximab experiencing IRRs, all of which were low grade, and no IRRs with placebo [36]. Preliminary clinical results for BCD-132 showed that 25.0% (6/24) of patients with MS treated with BCD-132 reported IRRs. Further studies are currently ongoing in larger and adequately powered cohorts to fully elucidate the risk of IRRs (Table 2) [40, 118].

Overall, the IRR data from these trials indicate a reduced incidence of IRRs with low-dose subcutaneous injection of ofatumumab compared with the intravenously infused mAbs [116]. It is thought that ofatumumab’s fully human molecular structure and the ability to administer the antibody at lower doses due to its direct targeting of lymph nodes via subcutaneous injection are major factors in ofatumumab’s reduced IRR incidence [41]. Although ofatumumab does induce IRRs, premedication has not been shown to significantly reduce this incidence, and it is therefore not required for administration [32, 97]. Conversely, premedication is required before each infusion of ocrelizumab, rituximab and ublituximab [41].

### Infections and hypogammaglobulinemia

The immune interference of anti-CD20 mAb therapies can diminish patients’ immune responses and increase susceptibility to infection [119], which may increase the already elevated risk of infection associated with MS [120].

Interestingly, similar rates of infection were reported for the placebo and rituximab groups in the phase II HERMES study of rituximab in patients with MS [52]. Moreover, previous phase II trials of ocrelizumab in patients with RRMS found no difference in the incidence of infection between the placebo and ocrelizumab groups [121]. Because both studies collected safety data at < 52 weeks, this may indicate that the risk of infection associated with anti-CD20 mAbs increases during long-term treatment. Consistent with this, the 96-week OLYMPUS trial of rituximab found that 4.5% of patients treated with rituximab experienced serious infections, compared with < 1.0% of patients receiving the placebo [53].

Long-term use of anti-CD20 mAb therapies is thought to reduce serum Ig levels [41], which is consistent with an increasing risk of infection over time. A longer-term assessment of patients treated with ocrelizumab over 6 years found that reductions in serum Ig levels were associated with an elevated risk of serious infection. Moreover, this association is stronger in relation to the levels of IgG, rather than IgM or IgA [122], suggesting that serum IgG levels may be an indicator of infection risk in patients treated with anti-CD20 mAb therapies. Consistent with this hypothesis, patients receiving ofatumumab for 3.5 years displayed stable levels of serum IgG, which corresponded with low incidence of serious infection. This is despite 23.1% of patients experiencing IgM levels below the LLN at least once during the trial [116].

During the HERMES clinical trial of rituximab, infections were reported in 69.6% (48/69) of patients treated with rituximab, with upper respiratory tract infections and urinary tract infections being among the more common infections reported, affecting 18.8% (13/69) and 14.5% (10/69) of patients, respectively [52]. Furthermore, rituximab is frequently used off-label in Sweden as a treatment for MS, and several studies of patients with MS report that infections were the most common AEs associated with rituximab [123–125].

Across the OPERA I and II trials of ocrelizumab, infections were reported in 56.9% (232/408) and 60.2% (251/417) of patients, respectively [56]. Pooled data from both trials highlighted that upper respiratory tract infections and urinary tract infections were common, affecting 15.2% (125/825) and 11.6% (96/825) of participants treated with ocrelizumab [56]. Direct comparison between the ocrelizumab and interferon beta-1a treatment groups in these trials shows that upper respiratory tract infections were more prevalent in the ocrelizumab group (15.2% vs. 10.5%). In contrast, urinary tract infections were less common in the ocrelizumab group compared with the interferon beta-1a group (11.6% vs. 12.1%) [56].

Infections were reported in 51.6% (488/946) of patients receiving ofatumumab during the ASCLEPIOS I and II trials, which is a relatively low incidence compared with clinical trials of rituximab in patients with MS [57]. Consistent with findings for other anti-CD20 mAbs, upper respiratory tract infections and urinary tract infections were common types of infection reported among patients treated with ofatumumab, albeit at a reduced incidence, affecting 10.3% of patients [57]. The ULTIMATE I and II trials of ublituximab in patients with MS reported a similar incidence of infection, affecting 55.8% (304/545) of patients. Respiratory tract infections and urinary tract infections were reported in 7.7% and 4.0% of patients, respectively [35].

### Opportunistic infections

Cases of opportunistic infections such as hepatitis B reactivation and progressive multifocal leukoencephalopathy (PML), caused by the John Cunningham virus, have been reported in patients receiving anti-CD20 mAb therapies [126–128]. In line with published findings when looking at severe COVID-19 outcomes [129], an analysis of patients with MS receiving varied treatments found that rituximab was associated with the highest rate of serious infection [123]. This study reported two cases of PML in patients with MS, one of whom was receiving rituximab. In both cases, the patient had recently started a new treatment regimen, having switched from natalizumab therapy. It was concluded that the cases of PML resulted from natalizumab treatment, which has previously been linked to PML [123].

The increased risk of infection associated with MS, the age of the patient and the immune suppression resulting from ocrelizumab treatment were all deemed to have contributed to a previously reported occurrence of PML [128]. However, a further nine cases of PML were reported in patients with MS during the post-marketing setting for ocrelizumab. Most of these cases were linked to prior treatment with natalizumab and fingolimod, with the exception of one patient who had not received prior treatment with disease-modifying therapies. This represented the first occurrence of PML directly associated with ocrelizumab treatment [130, 131]. A documented case of hepatitis B reactivation also occurred in a patient with MS receiving ocrelizumab [126]. Despite the absence of detectable hepatitis B viral DNA at the start of ocrelizumab treatment, reactivation of the infection was confirmed 6 weeks later [126]. The risk of infection reactivation should be considered in treatment decisions and can be mitigated through an effective program of screening and preventative treatment [132, 133].

### COVID-19

Given the links between anti-CD20 mAb therapy and an increased risk of infection, there was speculation over the susceptibility of patients with MS to severe COVID-19 infection [41]. A retrospective analysis of patients with MS in Italy reported an association between COVID-19 infections and rituximab and ocrelizumab, respectively. Moreover, these anti-CD20 mAb therapies were linked to more severe COVID-19 infections and increased frequency of hospitalization compared with other therapies [134]. Further retrospective analyses found that rituximab and ocrelizumab were associated with a higher risk of COVID-19 infection compared with alternative drug classes. However, this study did not find that these anti-CD20 mAb therapies meant patients were more prone to COVID-19-related hospitalization compared with alternative therapies [135]. A systematic review of severe COVID-19 outcomes found disparities in patients with MS receiving either rituximab or ocrelizumab [129]. A comparison of a range of MS treatments found a pooled estimate of death of 1.8%, while the estimate for patients receiving rituximab or ocrelizumab was 4.5% and 1.6%, respectively [129]. However, these findings did not consider the length of time patients had been treated with anti-CD20 mAb therapies [129, 134]. Retrospective analysis of North American patients with MS and COVID-19 found that rituximab was associated with a significant increase in the risk of hospitalization. Ocrelizumab treatment was also associated with an increase in the risk of hospitalization, although this association was less pronounced compared with rituximab [136].

The ALITHIOS trial of ofatumumab found that 8.2% (139/1703) of patients reported COVID-19 infections, of which 94.2% (131/139) were considered mild or moderate and 7.2% (10/139) were considered serious [137]. When compared with the general population, these findings suggest that ofatumumab did not increase the susceptibility of patients to severe COVID-19 infections [137]. This may be influenced by the stability of IgG serum levels associated with ofatumumab treatment [116], but further data and analyses are needed before this can be concluded.

The effects of anti-CD20 mAb therapies on COVID-19 vaccine response in patients with MS have also been a point of concern [41]. Given the relatively recent development of COVID-19 vaccines, there are limited findings related to the effect of anti-CD20 mAb therapies on vaccine response. Analysis of anti-CD20 mAb therapies found varied seroconversion following COVID-19 vaccination. Patients who received rituximab and ocrelizumab demonstrated seropositivity of 11% (1/9) and 43% (19/44), respectively [138]. Diminished humoral responses to a range of vaccines (not including the COVID-19 vaccine) were reported in patients with MS receiving ocrelizumab [139]. A retrospective study of patients treated with ocrelizumab reported a positive serological response in 37.5% of patients following COVID-19 vaccination. However, these findings were based on a small sample size, and the techniques used to collect serological data were inconsistent [140].

Seropositivity was demonstrated in 75% (3/4) of patients treated with ofatumumab [138]. However, the small sample size limits the conclusions that can be made from these data. Additional findings from patients with MS treated with ofatumumab have reported reduced humoral immune responses following COVID-19 vaccination, without significantly disrupting T-cell responses [141]. Likewise, Faissner et al. reported that patients with MS treated with ofatumumab for 3 months had significant depletion of B cells and an impaired humoral response following COVID-19 vaccination. Despite this, T-cell response remained strong and underpinned preserved cellular immunity [84].

### Malignancies

The immune suppression associated with anti-CD20 mAb therapies may increase the risk of malignancies developing in patients with MS [42]. A retrospective analysis of patients with MS in Sweden reported that rituximab did not increase the risk of invasive cancers, when compared to the general population [142]. Further, no incidences of neoplasm were reported in the ULTIMATE I trial of ublituximab; however, the incidence was 0.7% (2/272) during the ULTIMATE II trial [35]. The rates of neoplasm reported during the ASCLEPIOS I and II trials in patients with MS receiving ofatumumab were 0.5% (5/946) [57]. During the OPERA I and II trials, neoplasms were reported in 0.5% (4/825) of patients in the ocrelizumab group, with a further five cases of neoplasm detected during the open-label extension phase [56]. Of the nine cases of neoplasm reported, 44.4% (4/9) were a form of breast cancer [56]. However, subsequent analysis of safety data across multiple clinical trials and real-world sources found that the standardized incidence ratio for breast cancer for people treated with ocrelizumab did not indicate an elevated risk when compared with a typical population of patients with MS. Furthermore, analysis of real-world data found that the incidence rate of breast cancer was not elevated in patients treated with ocrelizumab compared to the US general population [130]. Cumulatively, these findings suggest that the associated risk of malignancies appears to be low across anti-CD20 mAb therapies.

### Pregnancy

Prescribing information for anti-CD20 mAb therapies advises against receiving this treatment during pregnancy, and that effective forms of contraception should be used when undergoing treatment [25, 31, 32, 37].

A retrospective analysis of pregnancies that featured rituximab use within 6 months of conception reported B-cell depletion in 39% of newborn babies. However, B-cell levels were restored within 6 months post-birth [143]. An additional study reported two cases of rituximab treatment at 13 and 21 weeks’ gestation, respectively, and observed no clinical or developmental adverse outcomes [144]. In patients with MS treated with ocrelizumab before pregnancy, congenital abnormalities were reported in 1.6% of live births, which is consistent with the 2.1% rate seen in the UK general population [145]. Moreover, a case study of a patient with MS treated with ocrelizumab at 19 weeks’ gestation reported normal neonatal lymphocyte levels at birth, followed by normal developmental milestones being met at 3 months of age [146]. Analyzing data from the ASCLEPIOS I and II, ALITHIOS, MIRROR and post-marketing trials of ofatumumab, no birth defects or congenital abnormalities were reported in 23 pregnant patients, and further observations and analysis of patients with MS exposed to ofatumumab during pregnancy are being planned [147]. Owing to limited data surrounding the effects of anti-CD20 mAb therapies in pregnant people, it is difficult to draw firm conclusions regarding safety. Observations from larger cohorts of pregnancies exposed to anti-CD20 mAb therapies are required.

### Colitis

Rituximab has previously been linked to gastrointestinal injury, such as diarrhea and bowel perforation. Moreover, there are multiple reports of colitis in patients treated with rituximab who had otherwise healthy bowels prior to starting treatment [148–150].

One case study of a 62-year-old patient with marginal zone B-cell lymphoma reports the development of severe abdominal pain and colon distension, which required partial removal of the colon to alleviate symptoms [149]. After a recurrence of lymphoma, the patient received further rituximab treatment, which was followed by repeated abdominal pain and a diagnosis of severe colitis. The patient’s symptoms deteriorated before requiring eventual proctectomy treatment [149]. An analysis of patients treated with rituximab who experienced diarrhea found that 4.6% (21/460) had confirmed colitis. Interestingly, there were no reports of colitis in the 47.6% (10/21) of these patients who had undergone a colonoscopy prior to starting rituximab treatment, suggesting an association between rituximab and the development of colitis [148].

Likewise, a case of treatment-emergent colitis was reported in a patient with MS treated with ocrelizumab who had no prior history of inflammatory bowel disease [151]. In this instance, colitis developed following only two rounds of ocrelizumab treatment and resulted in the patient undergoing a total colectomy [151]. Diarrhea has also been reported as an AE in clinical trials for ofatumumab and ublituximab [36, 57, 116]; however, it is unclear if these instances were due to colitis.

The risk of colitis may be underestimated because it is a rare and unrecognized event. Emphasis should be placed on studying the link between colitis and anti-CD20 mAb therapies, with the aim of reducing the risk of colitis and the necessity for significant colitis-related clinical interventions.

## Tolerability

The anti-CD20 mAbs used for the treatment of MS are generally considered to have good tolerability with low levels of serious AEs and study discontinuations [41]. As discussed previously, IRRs are the most frequently reported AEs associated with administration of anti-CD20 mAbs for MS therapy [41]. Clinical trial data for all of the mAbs included in this review (except BCD-132 for which data are currently lacking) have reported that IRRs are most frequent upon administration of the initial dose, with incidence decreasing with each subsequent administration [41]. IRRs associated with anti-CD20 mAb administration are generally tolerable, with most IRRs being mild to moderate in severity [41].

To manage this risk of IRRs, the utilization of premedication is mandated for intravenously administered anti-CD20 mAbs to reduce the frequency and severity of IRRs. Premedication for rituximab [25, 152, 153], ocrelizumab [31, 154] and ublituximab [35, 37] must be given approximately 30–60 min before each treatment infusion. The premedication for these antibodies consists of intravenous methylprednisolone (or equivalent corticosteroid) and an antihistamine (such as diphenhydramine). An antipyretic, such as paracetamol, is also often included as part of the premedication regimen [25, 31, 37, 153, 154]. In the first-in-human trial of BCD-132, patients were given premedication with methylprednisolone, paracetamol and an antihistamine (or equivalent medications) with further studies ongoing to assess IRR tolerability in larger cohorts [40, 118].

For ofatumumab, premedication is not required; however, healthcare professionals are instructed to inform patients that IRRs can occur, usually within 24 h of an injection and generally after the first injection [32, 97]. The nonessential nature of premedication for ofatumumab therapy was based on findings in the phase III ASCLEPIOS trials, which reported limited benefit in reducing the frequency of IRRs in patients who took premedication [57]. Therefore, premedication is not required, and instances of IRRs can be managed with symptomatic treatment if they occur [32, 97].

## Mode of administration: convenience and economic considerations

For chronic diseases such as MS, treatment burden upon a person’s life is becoming an increasingly important consideration within the context of decision-making by clinicians [155]. The biological and administration differences between anti-CD20 therapies for MS result in differing treatment regimens, which could have implications for treatment burden and health-related quality of life.

The approved regimens for rituximab (in rheumatoid arthritis) [25, 152, 153], ocrelizumab [31, 154] and ublituximab [35, 37] require them to be administered by healthcare professionals with access to medical support. These regimens also comprise infusions (and associated premedication and/or post-dose monitoring periods) that take place over several hours (Table 2). Less is known about the other afucosylated anti-CD20 mAb BCD-132, but the phase I trial by Boyko et al*.* reported study-site intravenous administration of BCD-132 over several hours to patients with RRMS at a range of doses (total dose of 100, 250, 500 or 1000 mg) [40, 156]. Patients either received the entire pre-set dose at a single visit or received the total dose over the course of two visits separated by 14 days, and all had pre-administered premedication [40]. Ofatumumab is unique among anti-CD20 mAb therapies because it is approved for use in RMS as a self-administered subcutaneous injection using either a pre-filled syringe or a Sensoready^®^ autoinjector pen following an initial self-injection under the guidance of a healthcare professional [32, 97]. However, a phase III clinical trial of subcutaneous ocrelizumab administered twice a year is ongoing and recently reported noninferiority for the primary endpoint, determined by pharmacokinetic measures of serum ocrelizumab levels over 12 weeks, compared to intravenous ocrelizumab in patients with either RMS or PPMS. Moreover, subcutaneous ocrelizumab was comparable to intravenous ocrelizumab in controlling magnetic resonance imaging lesion activity in the brain over 12 weeks [157, 158]. If approved, this would be the first subcutaneous anti-CD20 mAb therapy approved for both RMS and PPMS.

There is interest among patients, clinicians and the pharmaceutical industry in a move toward subcutaneous injection as the standard route of administration for mAb therapies, owing to improved patient compliance and reduced financial burden on healthcare systems [155]. Moreover, given the ongoing COVID-19 pandemic and the greater susceptibility of patients with MS to infection, reducing hospital visits has potential benefits in terms of infection risk management [159]. A previous study assessing adherence to injectable disease-modifying therapies in MS demonstrated that autoinjector use was the strongest predictor of treatment adherence at 24 months [160]. Systematic assessment of studies reporting patient preferences across therapy areas also demonstrated a preference for subcutaneous over intravenous administration [161].

Less is known about the implications of subcutaneous versus intravenous anti-CD20 mAb therapy administration on healthcare resources, specifically in the context of MS. However, a previous assessment of payer considerations reported distinct cost savings arising as a result of shifts from intravenous to subcutaneous administration [159]. Furthermore, an analysis of rituximab administration found that mean active healthcare professional time was reduced by 32% across oncology units when rituximab was administered subcutaneously [162]. A study in a Swedish hospital looking at the use of subcutaneous trastuzumab administration in patients with breast cancer reported significant time and financial savings. Compared with intravenous infusion, the subcutaneous administration of trastuzumab to 178 patients with a new diagnosis saved nurses 1101 h [163]. Moreover, analysis of cost differences illustrated a financial benefit of 603,000 EUR following a subcutaneous administration program [163]. The potential economic benefits of subcutaneous administration may go some way to offset the financial costs of ofatumumab, which are greater than the financial costs of rituximab and ocrelizumab, respectively [95].

These differences are important considerations for clinicians and patients as part of a shared decision-making process for a chronic disease such as MS. Distinct treatment regimens will result in different levels of clinical visits, treatment burden and convenience, flexibility and patient independence. Treatment choice should factor in these health-related quality-of-life considerations alongside efficacy, safety and tolerability discussions.

## Conclusions

Recent years have seen an exponential growth in our understanding of the integral role of B cells in the pathogenesis and pathobiology of MS. Our increased understanding in this field has seen the advent of an age of widespread regulatory body approvals for effective anti-CD20 mAbs for the treatment of people with MS.

However, despite targeting the same molecular target on pathological B cells, anti-CD20 mAbs have distinct molecular and biological characteristics that result in substantial differences in their clinical characteristics. These differences include varying efficacy, safety and tolerability, which stem from subtle differences in the antibodies’ mechanisms of action and routes of administration. Furthermore, potential differences in the convenience of use have important considerations for patients in relation to health-related quality of life and disease management, particularly pertinent for a chronic disease such as MS.

The current evidence shows that all anti-CD20 mAb therapies are well-tolerated and achieve near-complete depletion of B cells. Further ongoing clinical trials, especially those assessing the long-term and real-world use of anti-CD20 therapies in MS, will potentially shed further light on the similarities and differences between anti-CD20 therapies. Clinicians, alongside patients and their families, should take the aspects discussed in this review into consideration during the treatment decision-making process to help improve outcomes and health-related quality of life for people living with MS.
